# The Role of Psychological Time in Late Socialization. A SEM Analysis

**DOI:** 10.11621/pir.2023.0311

**Published:** 2023-09-30

**Authors:** Ekaterina V. Zabelina, Olga S. Deyneka, Yulia V. Chestyunina, Ekaterina V. Vedeneeva

**Affiliations:** a Chelyabinsk State University, Russia; b Saint-Petersburg State University, Russia; c Rostov State Technical University, Russia

**Keywords:** aging society, late socialization, psychological time, temporal focus, subjective age, subjective well-being, life satisfaction, retirees

## Abstract

**Background:**

Psychological time, a subjective reflection of the objective passage of time, has age specific characteristics and can be considered a resource for adaptation to difficult life situations (Pultz, & Hviid, 2016). We assume that the components of psychological time are also a resource for adaptation to retirement, smoothing out undesirable social and biological changes in retirees’ lives.

**Objective:**

This study explores this hypothesis by identifying the contribution of the cognitive component of psychological time — temporal focus and subjective age — to the effectiveness of late socialization.

**Design:**

The developed theoretical model was verified by the SEM method on the sample of retirees from Chelyabinsk, Russia (N = 291). To collect the empirical data we used the Temporal Focus Scale (Shipp et al., 2009), the Age of Me (Barak, 2009), the Life Satisfaction Scale (Diener & Lucas, 1999), the Income Satisfaction Scale (Deyneka, 2000), and questionnaire variables.

**Results:**

Temporal focus and subjective age mediated the influence of biological and social variables on the retirees’ subjective well-being. A younger subjective age smoothed the contribution of educational level, working status, and disability, whilst the current and future focuses mediated the association between religiosity and various parameters of satisfaction.

**Conclusion:**

The results of the study broaden the understanding of psychological time in the late socialization process. A pronounced focus on the present, along with younger subjective age, can be seen as psychological resources, allowing for better adaptation to the social status of a retiree; that is, increasing the effectiveness of late socialization.

## Introduction

The problem of an aging society and its consequences has until recently only been a concern of individuals, but since the second half of the twentieth century, the whole world community has become concerned about this process (Herath, 2017; Teixeira, Nagarajan, & Silva, 2017; Vogel, Ludwig, & Borsch-Supan, 2017). The contradiction between a person’s increased life expectancy, on the one hand, and the not always high social productivity and personal happiness experienced in retirement, on the other, is unresolved. Nevertheless, despite the joint efforts of scientists, politicians, and public figures in this field (https://www.garant.ru/news/1221705/), this problem is still not solved in Russia, and the means for its solution are not obvious.

One promising approach to the study of an aging society involves seeing it through the prism of late socialization (Zabelina, & Chestyunina, 2022), because that approach allows retirement to be seen as an opportunity for effective adaptation to a new way of life and self-realization in a new social environment. In social psychology socialization is viewed as a two-way process of individual absorption of social experience, a system of social ties and relationships, norms of behavior, social roles, and values which enable individuals to function successfully in society and achieve self-realization (in a social environment) (Andreeva, 1980). It is customary to distinguish between primary (early) and secondary (adult) types of socialization (Yantzen, 2015). Late socialization is understood as a type of secondary socialization (Drobysheva, & Zhuravlev, 2016), the process and result of a person’s rethinking his or her life and the surrounding reality, while adapting to a new social status (retiree), accompanied by changes in consciousness and behavior.

Psychological factors are important in late socialization, as they allow a person to use his own resources to adapt to the changes which accompany older ages (Sergienko, & Kharlamenkova, 2018), and help to dispel negative social stereotypes of older persons. For example, it was found that the older generation of Russian residents not only actively uses Internet gadgets, but do it more ethically compared to younger people (Maksimenko, Deyneka, & Dukhanina, 2022).

One of the non-obvious psychological factors influencing the adaptation to retirement is psychological time and its components (the perception and experience of time, attitude toward time, etc.). Since the passage of time is not perceived in the same way at different ages (Sircova et al., 2014), it can be assumed that its late-onset features may affect the success of adaptation to retirement. Since psychological time is a subjective reflection of the objective passage of time in the psyche, it is clearly amenable to correction by psychotherapeutic methods: that is, there is an opportunity to make retirement happier.

In spite of this fact, the role of psychological time in the process of adaptation to retirement has not been sufficiently studied. The question of how the perception of time and self-image in this period of life affects a person’s identity upon acquiring the status of pensioner is ambiguous. Moreover, research on psychological time in old age, including its cognitive component, generally focuses on clinical cases (*e.g.*, Balashova, 2016; Mikeladze, 2014) and does not always take into account the parameters of the norm. Thus, the purpose of our study was to identify the role of psychological time, namely its cognitive component, in the process of late socialization.

## Theoretical review

### Late socialization: criteria and factors

The socialization of older persons (late socialization) refers to the development of knowledge and skills, the adoption of new behaviors, and changes in values, all of which should ensure adequate adaptation to one’s age and appropriate participation in interaction with society (Dubynina, 2019). Related concepts include active aging (Cao, 2022; Zheng et al., 2023); successful aging (Martinson, & Berridge, 2015; Zhang et al., 2018); healthy aging (Scult et al., 2015; Arroyo-Quiroz et al., 2020); effective aging (Strizhitskaya, 2017); favorable aging (Sergienko, & Kharlamenkova, 2018); social adaptation/disadaptation (Prokhorova, 2022); desocialization (Belikov, 2019); and resocialization (Perinskaya, 2005; Serdakova et al., 2020). These concepts try to describe the effectiveness of socialization at a late age: how successfully a person can adapt in society when changing his or her lifestyle (retirement).

However, despite the variety of terms used to refer to late socialization, the research focus has been on objective criteria of socialization, such as activity and productivity in society (Pinto, & Neri, 2017), independence (Galinha, Pinal, & Lima, 2021), participation in work and social life (Pavlova, Gumennikov, Monastyrny, 2017), life quality (Yantzen, 2015; Galinha, Pinal, & Lima, 2021), level of material security (Drobysheva, & Zhuravlev, 2016), health (Pinto, & Neri, 2017; Westerhof, Miche, & Brothers, 2014), favorable environment (Belikov, 2019; Yantzen, 2015), and the like.

In contrast, our study focused on subjective criteria like life satisfaction and happiness (Pavlova, Gumennikov, & Monastyrny, 2017). We proposed considering various aspects of subjective well-being as criteria for effective late socialization, — namely, life satisfaction in general (Diener & Lucas, 1999), economic satisfaction (Deyneka, 2000), health satisfaction, and the modality of perception of life in retirement.

To date, a large number of factors (biological, social, psychological) influencing the process of late socialization have been studied (*e.g.*, Melekhin, 2016; Yantzen, 2015). While we understand the conventionality of this division of factors into groups, we used it to make the theoretical model as clear and transparent as possible. One obvious factor is health. There are objective reasons to highlight this factor, because some diseases (ischemic heart disease, hypertension, diabetes mellitus, respiratory and digestive diseases, diseases of visual and hearing organs, diseases of the central nervous system, and oncological diseases) are the companions of old age for most retirees (Vaganova-Naimushina, 2017). In this study, the presence or absence of disability status was chosen as a factor of late socialization reflecting objective health problems.

In a number of studies, the level of physical activity in old age is considered a factor influencing physical health (VOZ, 2010; Nicholson, 2004; Paterson, & Warburton, 2010) and late socialization as a whole (VOZ, 2010). However, according to research on the Russian sample, retirees’ physical activity is not realized through playing sports, but mainly through performing various types of work (paid work, gardening, childcare, fishing, hunting, etc.) (Zasimova, & Sheluntcova, 2014). Therefore, not only the state of health but also the indicator of working status was taken as a factor of late socialization in this study. In the scientific literature there is a lot of evidence of the positive impact of employment on the assessment of emotional state and psychological well-being of retirees (Konoplev, & Konopleva, 2016; Prokopets, 2017). The attitude toward online work of older employees is also being studied (Toscano et al., 2022).

Closely related to the possibility of continuing to work is the issue of the retirees’ level of education (Shakhmatova, 2021; Sergeeva, & Borisov, 2020), which can also be considered one of the factors contributing to life satisfaction in this period (Andreenkova, & Andreenkova, 2019). The presence or absence of a family (Bukhalova, 2011; Iakimakha, 2002) can also be one of the factors determining the process and the result of late socialization, and can lead to the development of the problem of loneliness at an older age (Naumova, & Glozman, 2021; Gekht, 2001).

A number of studies on gerontopsychology suggest that there is an increasing interest in religion as people age (Shangareeva, & Kozyrev, 2016; Kofanova, & Mchedlova, 2010). Increasing religiosity in old age gives strength, helps people to cope with the fear of death, and helps to foster humility in difficult life situations. Thus, religiosity can also be seen as a factor of late socialization in this study.

### Psychological time and its cognitive component

Psychological time as a subjective reflection of the passage of time in the human psyche has not yet been specifically studied in the process of later socialization; however, the prerequisites for this study exist. According to Kovalev (1988), psychological time is a “person’s perception and experience of his or her objective time of life, a concept of time conditioned by both personal experience of individual and group life, by one’s social, historical, and cultural experience, and by the person’s awareness of the passage of time, personal relationship to time, and psychological organization and regulation of vital time” (p. 217). The international scientific community presents a wide variety of terms to describe psychological time: temporal orientation (Holman & Silver, 1998); time attitude (Nuttin, 1980; Nestik, 2011, 2015); polychronicity (Bluedorn, 2000); temporal depth (Bluedorn, 2002); time perspective (Boniwell, & Zimbardo, 2004; Zimbardo, & Boyd, 1999); temporal focus (Shipp et al., 2009); MindTime (Zabelina & Fortunato, 2019); subjective age (Barak, 2009); time experience (Golovakha, & Kronik, 1984), and others.

According to Nestik’s (2015) model of attitudes toward time, psychological time has four components: 1) value-motivational (subjective value of time as an irreplaceable resource); 2) cognitive (temporal perspective, temporal aspects of identity); 3) affective-evaluative (the emotional relationship of personality to time); and 4) conjectural (preferred ways of organizing time) (p. 100). In our study, we focused on the cognitive component of retirees’ psychological time — subjective age and time focus — as the least studied and giving contradictory data.

A review of the theoretical frameworks used to study the problem of psychological time in old age as a factor of late socialization revealed differences in the approaches of foreign and Russian authors researching this issue (Danilova, & Zabelina, 2022). The latter tend to consider psychological time of the elderly from a clinical-psychological standpoint, analyzing its cognitive deviations (Balashova, 2016; Mikeladze, 2014; Bragina, 2013; Melyohin, & Kireeva, 2016; Metsler, 2015). The research by foreign authors follows three main directions: 1) a future time perspective (Demiray, & Bluck, 2014; Daly, Hall, & Allan, 2019; Korff, Biemann, et al., 2020); 2) subjective age (Blöchl, Nestler, & Weiss, 2021); and 3) perspective on the past (Holman, & Silver, 2016; Mello, Barber, & Vasilenko, 2022). However, there is plenty of contradictory data within the domains of psychological time research on old age, both in Russia and abroad. Some phenomena, such as the temporal focus of older people, have been left out of the international research. This confirms the need for further study of psychological time, especially its cognitive component, on the Russian sample of mentally healthy elderly people in retirement.

Temporal focus is a person’s degree of attention to various aspects of life, which can concentrate on their past, present, or future (Shipp et al., 2009). While it is a stable feature, temporal focus nevertheless is still subject to fluctuations during one’s life. As a person ages, he or she realizes that life is finite, and focuses less and less on the future (Carstensen et al., 1999). In addition to natural adulthood, there may be significant events (Shipp, Aeon, 2019) that affect temporal focus, and retirement may be one of them.

A person’s subjective age is the self-perception of one’s own age (Sergienko, 2020). Research on the relationship between subjective age and subjective well-being in old age results in contradictory data. Most scholars conclude that feeling younger (younger subjective age) has a positive effect on life satisfaction in old age (Blöchl; Nestler, & Weiss, 2021). However, some studies (Veenstra, Daatland, & Aartsen, 2021) have found that the desire to be younger is often related to dissatisfaction with life and poor physical health. These data deficits and inconsistencies have led to the inclusion of temporal focus and subjective age as cognitive components of psychological time as factors of late socialization by Nestik (2015) in his theoretical model.

### A priori model

**Figure 1.**
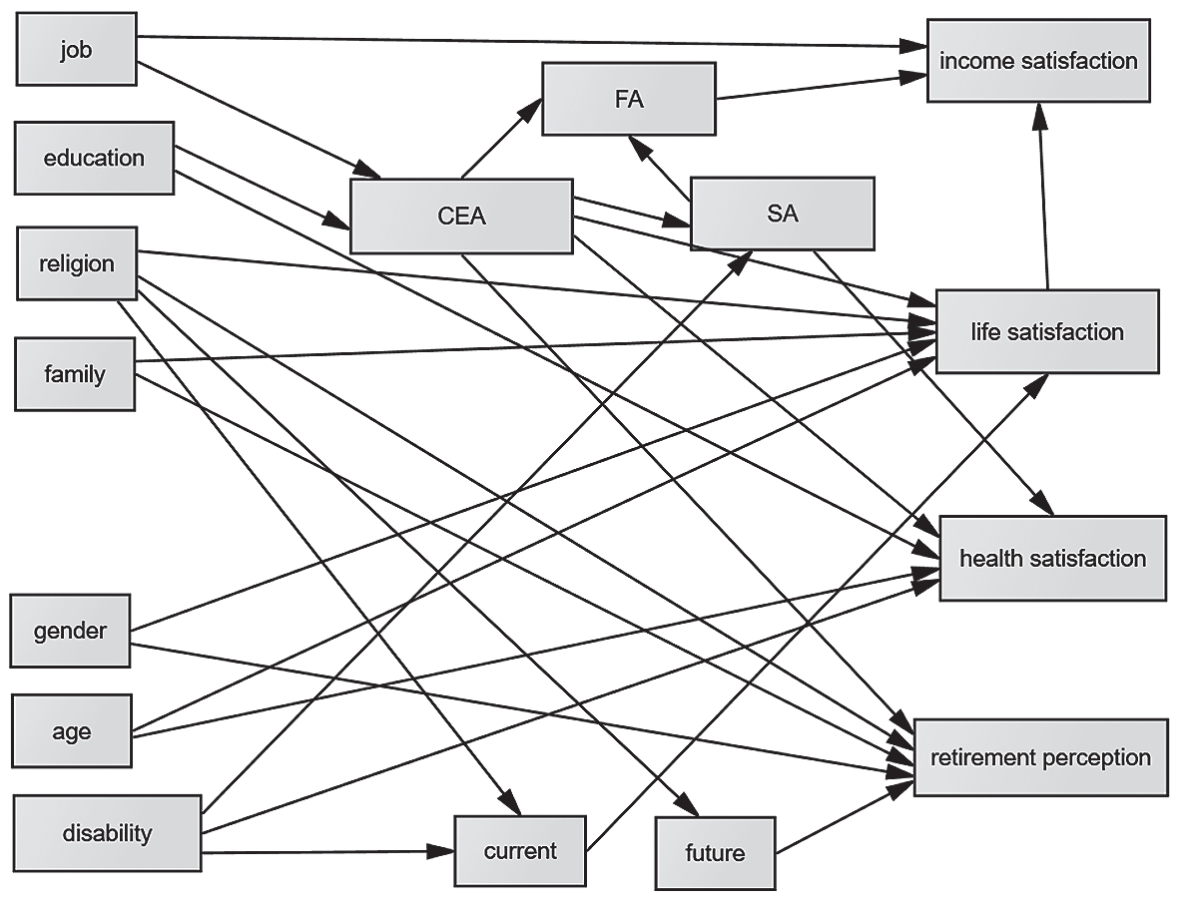
A priori model of contribution of psychological time to late socialization

*Notes: CEA = cognitive-emotional age; SA = social age; FA = physical age; current = current focus; future = future focus. Biological factors: gender, age, disability. Social factors: job, education, religion, family.*

Theoretical analysis allowed us to construct a model of the contribution of psychological time to late socialization (*Figure 1*).

The model we developed consists of several components (factors and criteria of late socialization), wherein psychological time serves as the mediator of the relationship between the factors of socialization (biological and social) and the criteria of its effectiveness (subjective well-being). By the effectiveness of late socialization, we mean the degree to which an older person is able to adapt to the new social status of a retiree. Within the framework of this model, we use only the internal (subjective) criteria of efficiency, because today more and more weight is given to subjective assessments of the human condition compared to the objective (*e.g.*, Westerhof, Miche, & Brothers, 2014).

For example, in medicine, a person’s perception of their disease is considered more relevant than the objective indicators of disease (Vasserman, Chugunov, & Shchelkova, 2019). It is believed that only the person knows best what is good or bad for him. Besides, according to some research (Dubynina, 2019), while other stages of socialization are associated with such solemn rituals as the graduation ceremony, wedding, etc., in old age, life itself comes to the forefront of a person’s inner sense of identity.

Biological and social factors (conditional separation) within this model are considered as independent variables, because they are effectively given to the person from the outside, — that is, defined objectively, they are difficult to influence by one’s desire. Social factors in this case are the factors that trigger change and the need to socialize anew. For example, retirement often involves the termination of a professional career, the destruction of some social (working) ties, etc.

Psychological time acts as a subjective factor, because by mediating external (objective) events, psychic perception can change a person’s interpretation and attitude toward them. That is why we assume its mediating role in the model.

Finally, subjective well-being in retirement seems to be an even more subjective factor that is likely to change in response to changes in external social conditions, taking into account the individual’s perception. Subjective well-being, or satisfaction with various aspects of life, is most susceptible to change in response to changes in the retiree’s environment (*e.g.,* Shchukina, & Shirman, 2022); thus, we consider it as a dependent variable. The a priori model was then verified in our empirical study.

## Methods

Our research methods included questionnaires to collect data on social and biological factors of late socialization. The biological factors identified were the gender of the respondents (1 = male, 2 = female), their chronological age, and whether they had a disability (1 = yes, 2 = no).

The following were identified as social factors: a) level of education (1 = primary, 2 = general secondary (school), 3 = secondary vocational, 4 = higher); b) family (1 = married, 2 = single, 3 = divorced, 4 = widowed); c) working status (1 = working, 2 = not working); and d) the level of religiosity (1 = atheist, 2 = indifferent to religion, 3 = admit the existence of the Higher Forces, 4 = I am a believer, 5 = I believe and try to observe the rites of my religion).

For diagnostics of temporal focus we used the Russian version of the Temporal Focus Scale (Shipp et al., 2009). The technique is a simple tool which includes 12 statements and 3 subscales: 1) focus on the past (M = 5.21, SD = 1.46); focus on current events (M = 5.17, SD = 1.17; and focus on the future (M = 4.44, SD = 1.57). The scale showed a high level of reliability on the Russian sample (α = 0.879).

To explore subjective age, the Age-of-Me scale was applied (Barak, 2009) (α = 0.913). The questionnaire consists of four statements with missing values, in which a digit must be entered: 1) I feel __ years old; 2) I think I look ___ years old; 3) In my opinion, I act like a person of __ years; and 4) My interests mainly correspond to the interests of a person __ years old. The first statement characterizes the cognitive-emotional age (feeling-age), the one at which a person “feels oneself ” (M = 54.6, SD = 15.38). The second statement reflects the biological (physical) age (look-age) — “the age that a person looks like” (M = 57.9, SD = 12.57). The third statement conveys social age (do-age) — “the age at which a person acts” (M = 56.7, SD = 12.51). The fourth statement describes the intellectual age of a person (interest-age); this is “the age at which a person shows his or her interests” (M = 56.87, SD = 11.79).

The results were counted in two ways. First, the figures (age) given by the respondents in the survey (on four scales) were the primary data. Second, the difference between the age indicated in the statements by the respondents and the person’s actual chronological age was calculated. In the second case, the higher the indicator, the younger the subjective age is diagnosed.

The main criterion for late socialization — the subjective well-being of the retirees — was diagnosed with several indicators: 1) Life satisfaction as a component of subjective well-being (Diener & Lucas, 1999); 2) Subjective assessment of income as an indicator of subjective economic well-being (Deyneka, 2000); 3) Subjective assessment of physical condition as health satisfaction; and 4) General perception and attitude toward retirement as a qualitative indicator of the satisfaction of life in retirement.

The level of happiness and subjective well-being was investigated with the help of the questionnaire by Diener and Lucas (1999). The Subjective Happiness Scale (M = 4.05, SD = 1.49) measures the emotional experience of an individual’s life as a whole, reflecting the overall level of psychological well-being. The main advantage of this method is its compactness, as well as its validity and reliability (α =0.874), thanks to a simple and unambiguous internal structure.

For the diagnosis of subjective economic well-being, the study used Furnham’s scale of subjective income level as adapted by Deyneka (2000). Respondents were asked to rate their income on a 7-point scale, with 1 being making ends meet and 7 being very high. This method (M = 3.56, SD = 0.96) is widely used in studies of economic psychology as an indirect indicator of a person’s subjective economic well-being.

Subjective assessment of the level of health was carried out using the authors’ scale of assessment of the physical condition of the respondents (M = 3.12, SD = 0.73). The evaluation was conducted using a 5-point Likert scale when answering the question “Assess your physical condition” (1 = very bad, 2 = bad, 3 = satisfactory, 4 = good, 5 = excellent). Thus, the higher value of this indicator in the model corresponds to a higher level of satisfaction with one’s health.

The diagnosis of general perception and attitude towards retirement was carried out using the method of open questions. Respondents were asked to complete the sentence: “Life in the retirement is...”. Their answers were subject to a thematic analysis (Braun, & Clarke, 2006), which allowed us to distinguish three semantic categories: 1) negative perception of retirement (*e.g.,* “sadness,” “idleness,” “death”); 2) neutral attitude to retirement life (*e.g.,* “another life,” “another stage in life,” “continuation of the path”), and 3) positive perception of retirement (for example, “dream,” “rest,” “live for yourself,” “freedom,” etc.). That is, the higher value of this indicator in the model corresponds to greater satisfaction of life in retirement.

The main mathematical method was structural equations modeling — SEM ( Byrne, 2016). Data processing was carried out using the statistical package IBM SPSS Statistics Rus. 24.0, including the AMOS module. To measure the model’s compliance with the original data, the method of structural equations modeling was used with recommended values of criteria: p — criterion significance level, X^2^ > .05, CFI > .95, RMSEA < .05, GFI > .9, PCLOSE = .5.

### Participants

The sample was randomly drawn from retirees living in Chelyabinsk (Chelyabinsk region, Russia) and included people with various living conditions (living separately, in families, in nursing homes, etc.), types of occupations, education levels, family relationships, and working status. The Chelyabinsk region is one of the largest economic units of the Russian Federation. The region’s industrial development is determined by the metallurgical, engineering, fuel and energy, construction, and agro-industrial sectors. The steel sector leads the economy, with more than 60% of the area’s industrial production. Since the majority of the population is employed in the metallurgical sector, many are likely to have health problems and to retire immediately after reaching the relevant age, except for employers and managers. There are some institutions of late socialization in Chelyabinsk, such as retiree clubs, libraries for the elderly, volunteer programs, etc. (http://chelib.ru/sitemap/to-readers/clubs/for-elders/). Many retirees have private plots of agricultural land.

A total of 291 retirees were surveyed (*Table 1*). The criterion for inclusion in the sample was having had the status of the retiree for at least one year, as well as the absence of a psychiatric diagnosis (clinical norm). Accordingly, the criterion for exclusion from the sample was non-achievement of retirement age, and not having the status of retiree.

**Table 1:** Sample characteristics

№	Respondents	Gender		N
		Male	Female	
1	Total	75	216	291
2	Employed	42	92	134
3	Unemployed	33	124	157
4	Disabled	15	60	75
5	Without disability	26	190	216
6	Married	50	106	156
7	Single	25	110	135

The number of women in the study sample is much higher than the number of men, because, according to ROSSTAT (https://rosstat.gov.ru/storage/mediabank/Bul_chislen_nasel-pv_01-01-2022.pdf), women live longer than men in general. Employed (N = 134) and unemployed (N = 157) retirees were represented in approximately equal proportions. Thirty-four percent (34%) of the total sample had a disability (n = 75), which is also natural, since the number of chronic diseases and other health problems resulting in disability increases with age. Also. the sample contained approximately the same number of single (n = 135) and married retirees (n = 156). Thus, it can be concluded that the sample is representative and reflects the general population as far as the main indicators are concerned.

Because older respondents may lose cognitive function (*e.g.*, loss of attention), the questionnaires were offered in paper form, and completed individually under the supervision of the researcher.

## Results

First, we tested the model to include all the variables that were being diagnosed (Byrne, 2016). In this case, we found some of the factors had no significant associations: gender (p = .094), age (p = .889), and family status (p = .129). The model’s fit was also far from acceptable: CMIN = 320.062, df = 95, p = .000; GFI = .878; CFI = .716; RMSEA = .090; Pclose = 0.000. Therefore, we decided to remove a range of indicators from the final model as not having sufficient weight to explain its content. After the removal of gender, chronological age, and family status, the model’s parameters improved significantly: CMIN = 114.127, df = 56, p = .000; GFI = .929; CFI = .865; RMSEA = .074; Pclose = .005.

**Figure 2.**
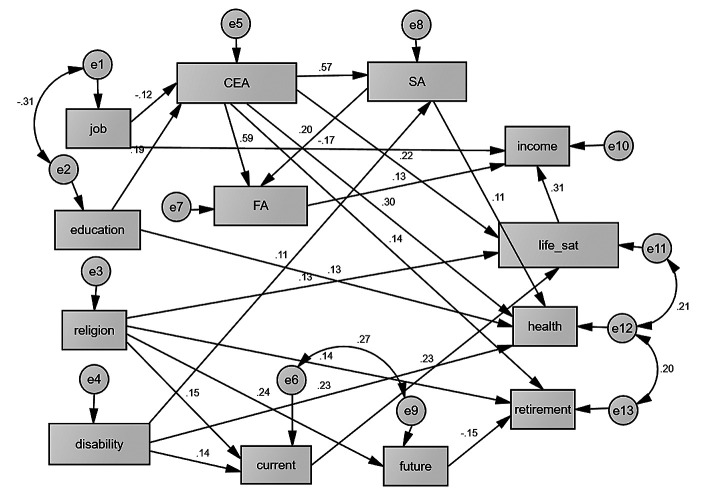
Posteriori model of the contribution of psychological time to late socialization

*Notes: CEA = cognitive-emotional age; SA = social age; FA = physical age; current = current focus; future = future focus; retirement = perception of retirement; life_sat = life satisfaction.*

In order to further improve the model’s suitability, we combined the errors of the variables of working status and education level, current and future focus, and three satisfaction indicators. The final model showed satisfactory correspondence indexes of the empirical data with the theoretical model (CMIN = 65.740, df = 52, p = .095; GFI = .967; CFI = .979; RMSEA = .030; Pclose = .944) (see *Figure 2*). All model elements showed significant associations (*Table 2*).

**Table 2:** Regression coefficients in the model

			Estimate	S.E.	C.R.	P
CEA	<---	job	–.895	.433	–2.064	.039
CEA	<---	education	3.579	1.143	3.131	.002
current	<---	religion	.179	.069	2.585	.010
current	<---	disability	.461	.186	2.483	.013
SA	<---	CEA	.430	.036	12.009	***
SA	<---	disability	3.139	1.362	2.305	.021
life_sat	<---	current	.297	.069	4.306	***
future	<---	religion	.384	.093	4.147	***
life_sat	<---	CEA	.024	.006	3.934	***
life_sat	<---	religion	.200	.083	2.401	.016
FA	<---	CEA	.443	.038	11.801	***
FA	<---	SA	.197	.050	3.931	***
income	<---	job	–.087	.028	–3.092	.002
health	<---	education	.138	.052	2.662	.008
health	<---	SA	.008	.004	1.885	.059
retirement	<---	future	–.078	.030	–2.584	.010
income	<---	life_sat	.198	.035	5.673	***
health	<---	CEA	.016	.003	4.753	***
retirement	<---	CEA	.008	.004	2.361	.018
retirement	<---	religion	.121	.049	2.460	.014
income	<---	FA	.013	.005	2.438	.015
health	<---	disability	.475	.101	4.685	***

*Notes: CEA = cognitive-emotional age; SA = social age; FA = physical age; current = current focus; future = future focus; retirement = perception of the retirement; life_sat = life satisfaction.*

The analysis of the posteriori model generally confirmed the basic assumption: the level of subjective well-being in retirement age is influenced by biological and social factors, while psychological time (its cognitive component) mediates this effect.

The presence or absence of disability was the only biological factor that showed a link with the criteria of late socialization. Disability had a moderate influence on the retiree’s subjective assessment of her health, both directly and through subjective (social) age, which mediates this impact. The absence of disability in old age increased the probability of assessing oneself as a healthier person, contributed to the shaping of interests of “younger age,” and allowed the retiree to feel included in a social group of younger people, which in turn reduced the risk of poor health scores. In addition, the lack of disability status contributed to overall retirement satisfaction indirectly through a focus on the present.

At the same time, the focus on current events and interest in them contributed to increasing life satisfaction. Thus, the analysis of the model revealed a significant influence of the biological factor — disability — on the effectiveness of late socialization, both directly and indirectly, mediated by social age and current focus.

Several social factors were important for the effectiveness of the late socialization process: the continuation of a professional career (working status), the level of education, and the level of religiosity. Although the contribution of each of these indicators was less than that of the biological factor of disability, collectively they were serious predictors of subjective well-being in retirement. In this case, elements of psychological time (temporal focus and subjective age) could adjust the influence of these factors.

Thus, the working status of a retiree may increase or decrease his or her satisfaction with their income (subjective economic well-being), depending on whether this influence is mediated by subjective age. In general, non-working retirees were more likely to experience lower income satisfaction, but for those who felt younger (cognitive-emotional and physical age), on the contrary, income satisfaction increased. It is likely that people of retirement age who perceive themselves to be younger than their age, have a positive attitude toward their age, maintain themselves in good physical and mental form, and extend this positive attitude to the assessment of their material condition. On the other hand, it is possible that older persons who invest in their physical and emotional well-being may feel more financially wealthy, regardless of their working status.

A similar pattern can be observed with regard to education. High educational level by itself did not directly affect income satisfaction, but, combined with younger subjective age (cognitive-emotional and physical one), contributed to greater subjective economic well-being. In addition, the feeling of being younger than one’s age contributed to a positive (optimistic) perception of life in retirement, increased self-esteem in health, and overall life satisfaction. That is, a younger subjective age served as a mediator to enhance the social factors of late socialization.

In addition, higher levels of education alone made a small contribution to the assessment of physical well-being in retirement (health), which can be attributed to greater knowledge about health and medicine, and a clearer understanding of where to fill knowledge gaps in this field. Education and the habit of constant learning can create a mindset of individual awareness, including in relation to one’s own health, which implies a more attentive approach to one’s physical condition and timely prevention of illness.

The level of religiosity of an elderly person, one’s faith in God and religious rituals, contributed to effective late socialization (satisfaction with life and the positive perception of life in retirement), both directly and indirectly — through the retiree’s temporal focus. Involvement in the events of the present (current focus) significantly increased this influence, while thoughts and worries about the future (future focus), on the contrary, negated this influence, worsening expectations about life in retirement (such as the time of death, boredom, loneliness, etc.). That is, despite the belief in higher forces, a focus on the future in retirement age may reduce the effectiveness of the process of late socialization.

## Discussion

The model we developed clarifies the role of psychological time (specifically, its cognitive component) in the process of late socialization by matching and complementing previous data. The impact of a focus on the present on life satisfaction has been confirmed by a number of studies (Shipp et al., 2009). However, in our study we have established the status of a current focus as the mediator between biological (disability) and social (religiosity) predictors of late socialization. Involvement in current events, interest in them, and motivation to actively participate in them, even despite objective health constraints, seems to be a recipe for happy old age.

It seems to be important to identify the fact that a pronounced future focus in retirement age may reduce the effectiveness of late socialization through the negative perception of the retirement lifestyle. Despite the novelty of the data obtained, they are generally consistent with Carstenen’s concept of a shorter time horizon for the future in old age (Carstensen et al., 1999). Most likely, anxiety about retirement, especially in the later period, leads people to look into the future more often, presenting it in negative terms.

Younger subjective age contributed to an increase in all diagnosed indicators of effective late socialization, as confirmed by other studies (Blöchl, Nestler, & Weiss, 2021; Sergienko, 2020). At the same time, the understanding that certain subjective ages may mediate (increase or decrease) the influence of other factors (*e.g.*, educational level, working status) broadens our understanding of the role of this phenomenon in the process of late socialization. Age perception seems to be the result of a complex interaction of various factors, objective and subjective, which needs further research. Moreover, the discovery of the role of different types of subjective age (physical, cognitive-emotional, and social) can be considered an outcome of the study, which could start a new line of research.

The role of objective health and disability in shaping the subjective well-being of the retirees, as noted in a number of studies (VOZ, 2010; Nicholson, 2004; Paterson, & Warburton, 2010), has also been confirmed in this model. The results of the study clarify the mechanism of this influence, which can be mitigated by social age and focus on current events. It can be assumed that retirees’ interest in modern trends, which can be realized thanks to IT technologies, allows older people to compensate for objective health problems and thus increases their subjective well-being.

In general, the model of psychological time as a factor shaping late socialization fits well with modern models of successful aging (Shchukina, & Shirman, 2022). To ensure the continuity of the personal development of older persons, it is necessary for them to continue to lead an active creative and social life, to reflect on their life experience, to rethink it creatively, and to use it in their current life situation.

## Limitations

The main limitation of the study was its narrow approach to socialization and its criteria as purely subjective (psychological). External criteria of socialization, such as the ability to function independently, productivity as a member of society and others, were not considered. On the other hand, the introduction of additional criteria would have complicated the already loaded model, causing possible computational difficulties. In addition, the research is impoverished by the fact that it did not consider the process of socialization, but only its outcome, and that only in a subjective way.

The study identified the role of only two cognitive components of psychological time. In further studies it is proposed to test the assumption about the role of nostalgia, time attitude, and time perspective in the process of late socialization. The study involved retirees living in one city with a strong industrial profile, which could have skewed the results. In the future, it is planned to expand the sample to include representatives from cities and towns in Russia with different urban orientations.

In addition, the study was limited in its methods of data collection. For example, the measure of disability can be more variable, including various degrees and nosologies that can determine a person’s satisfaction to some extent. In this study, we only accepted the diagnosis of disability without specifying the criteria, which makes the measurement quite crude. Moreover, some important biological and social indicators — namely, gender, age, and family status — may have a significant impact on how people live in old age but are not reflected in the empirical model. We explain this with a large number of variables in the model, where it is possible to “displace” less significant factors. A confirmation of this assumption can be found in other studies where the relationship of life satisfaction to the individual’s family situation is not determinative (*e.g.,* Shchukina, & Shirman, 2022). Further research with other design options is needed to clarify this finding.

Our model explains the causal relationships between factors and results of late socialization unilaterally. But it must be understood that psychic reality is always more complex than a theoretical model. Probably, in reality, the associations of the model elements are bidirectional; that is, they are mutually deterministic. For example, feeling younger than one’s chronological age (a younger subjective age) can increase subjective satisfaction with life. At the same time, it is possible that improved physical indicators due to rest or treatment may cause one to feel younger.

## Conclusion

Thus, the study succeeded in developing and verifying a model of the contribution of psychological time (its cognitive component) to late socialization. It was shown that temporal focus and subjective age are the factors that mediate the association between biological and social variables, and the subjective well-being of the retirees. In particular, it has been established that a younger subjective age serves as a mediator that strengthens the social factors of late socialization (level of education, working status), along with biological factors (presence of disability). The temporal focus mediates the contribution of religiosity to the effectiveness of late socialization: involvement in current events (current focus) greatly enhances the subjective well-being of retirees, while thoughts and worries about the future (future focus), on the contrary, offset this influence, worsening expectations for retirement.

Our results broaden the understanding of the late socialization phenomenon, determining time-cognitive predictors of its success. New evidence about the role of cognitive elements of psychological time in the process of late socialization provides the basis for the development of special training and counselling programs on the prevention of fear of aging, helping retirees acquire the ability to adapt to their age and increase satisfaction with later life. It is recommended that individuals, family systems, and community organizations all work to maintain older persons’ interests, stress their importance, and offer them an active role during retirement.

A separate niche in psychological practice with retired clients can be a discussion of their image of the future and perception of their own age. It is likely that changing their picture of the future and creating a more positive perception of themselves in it, as well as enrichment with modern interests, will make it easier for retirees to adapt to their new status and make this period of life happier, reducing the fear of aging and loneliness. We also recommend that preventive work be initiated before retirement age in preparation for late socialization. The study contributes to science by demonstrating the role of psychological time in enhancing the effectiveness of late socialization, suggesting a cognitive-therapeutic approach to overcoming the negative consequences of an aging society.
